# Calcium ion-induced formation of β-sheet/-turn structure leading to alteration of osteogenic activity of bone morphogenetic protein-2

**DOI:** 10.1038/srep12694

**Published:** 2015-07-27

**Authors:** Wenjing Zhang, Hongyan He, Yu Tian, Qi Gan, Jing Zhang, Yuan Yuan, Changsheng Liu

**Affiliations:** 1 The State Key Laboratory of Bioreactor Engineering, East China University of Science and Technology, Shanghai 200237, PR China; 2 Key Laboratory for Ultrafine Materials of Ministry of Education, East China University of Science and Technology, Shanghai 200237, PR China; 3 Engineering Research Center for Biomedical Materials of Ministry of Education, East China University of Science and Technology, Shanghai 200237, PR China

## Abstract

Preserving bioactivity of bone morphogenetic protein 2 (BMP-2) still remains a challenge in protein-based therapy. It is not known how Ca^2+^ released from extracellular matrix or existing in physiological environment influences bioactivity *in situ* till now. Here, effects of extracellular Ca^2+^ on conformation and osteogenic bioactivity of recombinant human BMP-2 (rhBMP-2) were investigated systematically. *In vitro* results indicated that Ca^2+^ could bind rhBMP-2 rapidly and had no obvious effect on cell behaviors. Low concentration of Ca^2+^ (0.18 mM) enhanced rhBMP-2-induced osteogenic differentiation, while high Ca^2+^ concentration (>1.80 mM) exerted negative effect. *In vivo* ectopic bone formation exhibited similar trend. Further studies by circular dichroism spectroscopy, fluorescence spectroscopy, together with cell culture experiments revealed at low concentration, weak interaction of Ca^2+^ and rhBMP^-^2 slightly increased β-sheet/-turn content and facilitated recognition of BMP-2 and BMPRIA. But, high Ca^2+^ concentration (>1.8 mM) induced formation of Ca-rhBMP-2 complex and markedly increased content of β-sheet/-turn, which led to inhibition binding of rhBMP-2 and BMPRIA and thus suppression of downstream Smad1/5/8, ERK1/2 and p38 mitogen-associated protein kinase signaling pathways. Our work suggests osteogenic bioactivity of BMP-2 can be adjusted via extracellular Ca^2+^, which should provide guide and assist for development of BMP-2-based materials for bone regeneration.

Segmental bone loss caused by osteoporosis, trauma, cancer, and congenital abnormalities remains a challenge for clinicians. Osteogenic growth factors that activate endogenous repair mechanisms can potentially regulate new bone formation and bone turnover. Of these growth factors, bone morphogenetic protein-2 (BMP-2), a member of the transforming growth factor (TGF)-β superfamily of multifunctional cytokines, has been shown to regulate osteoblast differentiation of cartilage and bone in the body, and has been widely applied for bone regeneration. Various matrix, including collagen gels^1^ (already used clinically), calcium phosphate salts^2^,^3^,^4^ and biodegradable polymers^5^,^6^,^7^,^8^,^9^, have been and are currently being adopted to carry recombinant human BMP-2 (rhBMP-2). However, many previous investigations found that the hydrogen/ionic/hydrophobic interaction with the delivery systems, and the environmental factors^10^ like pH, ionic strength, temperature, electrolytes, etc often led to the changes in secondary/tertiary structure and denaturation of rhBMP-2 *in vitro* and *in vivo*, which directly undermined its therapeutic efficacy and thus resulted in high dosage. Therefore, a better understanding and control of rhBMP-2’s conformation and the osteogenic bioactivity during its application *in vitro* and *in vivo* would be very significant to design and fabrication of the rhBMP-2-based orthopedic implants/scaffolds.

Currently, cations have been found to act as modulators of the secondary structure, stability, and biological activity of proteins. For example, S100A13, human S100B, and S100A12, three representative members of the S100 protein family, are stabilized by Ca^2+^ ions^11^,^12^,^13^. Conantokins-G, a potent antagonist of N-methyl-d-aspartate receptor channels, undergoes a structural transition to a helical conformation in the presence of multivalent cations including Mg^2+^ and Ca^2+^ 14. This complex formation could also induce the antiparallel dimerization of two peptide helices in the presence of Ca^2+^ but not Mg^2+^. Also, such effects of cations varied with different proteins. For instance, similar concentrations of Zn^2+^ ions could improve the stability of porcine S100A12, but had an opposite effect on S100A2^15^,^16^. Furthermore, just as the “hormesis” phenomenon observed in a variety of organisms, some trace elements, such as La^3+^, improved the catalytic activity of horseradish peroxidase (HRP) at low concentrations, but undermined its catalytic activity at high concentrations. Inspired by these above reports, the cations existed *in vitro* or *in vivo* or released from the extracellular matrix may exert effects on the conformation and osteogenic activity of BMP-2. To the best of our knowledge, there is no study to date focusing on this cation-induced changes in conformation and bioactivity of BMP-2.

Considering the fact that Ca^2+^ is the most pervasive component of the bone matrix/scaffold and is abundant in cell culture medium and the physiological environment, we investigated the effect of the calcium ion (Ca^2+^) on the conformation and osteogenic capacity of rhBMP-2 in this study. Conformational changes in rhBMP-2 were investigated using far-UV circular dichroism and steady-state fluorescence spectroscopy. The osteoblastic differentiation capacity of BMP-2 was studied by the typical osteoblast-specific marker, alkaline phosphatase activity (ALP) and osteogenic gene *in vitro* and the bone regeneration efficiency *in vivo*. C2C12, a pluripotent skeletal muscle myogenic progenitor cell line, which can differentiate toward an osteoblastic lineage in the presence of BMP-2 and thus is considered as a useful model for analyzing both the common and specific signaling mechanisms of BMPs, was chosen as a model cell line^17^. In *in vivo* bone regeneration evaluation, rhBMP-2 and Ca^2+^/rhBMP-2 were loaded into the FDA-approved gelatin sponge and were carried out in a mouse hind limb muscle pocket model using X-ray imaging and histological analysis.

## Results

In this study, to exclude the influence of the cations (1.8 mM of Ca^2+^ and 0.8 mM of Mg^2+^) in the culture medium, a specific DMEM without Ca^2+^ and Mg^2+^ was applied during the interaction of rhBMP-2 and cells. Considering the fact that BMP-2-induced osteogenic differentiation (including alkaline phosphatase activity (ALP), etc.) is often quantitatively evaluated after 72 h culture, a two-stage cell culture process, first with rhBMP-2-containing specific DMEM followed by the normal DMEM without rhBMP-2, was applied to circumvent any adverse effects of Ca^2+^-/Mg^2+^-free medium on cell viability and morphology over such extended period. In order to determine the specific time exposure to BMP-2-containing specific DMEM, we first studied the change of the ALP with the elongation of cultivation time with BMP-2-containing specific DMEM (the total culture time was fixed at 72 h). The results indicated that rhBMP-2, in the absence of cations, induced osteogenic differentiation in a time-dependent manner in the initial 12 h. An obvious increase in ALP activity was observed after 2 h interaction with rhBMP-2 and the highest ALP activity detected after a 6 h exposure (Supplementary Fig. S1a). No additional increase was observed with longer culture times. This increase in ALP activity was not due to the cell proliferation, since cell viability and morphology were unaffected in the two-stage culture process (Supplementary Fig. S1b,c). Therefore, in the following section, C2C12 cells were exposed in rhBMP-2-containing specific DMEM medium in the first 6 h and thereafter cultured in regular DMEM for another 66 h or 162 h for evaluation of the Ca^2+^ effect on the osteogesis of C2C12.

In the preliminary experiment, we found that with the above two-stage cell culture process, the OD value of ALP was within 0.5–1.0 when the rhBMP-2 level was in the range of 1.0–3.0 μg/mL, thereafter, the rhBMP-2 level was fixed at 2.0 μg/mL. Considering that the concentration of Ca^2+^ in normal culture medium is 1.80 mM, the extracellular calcium concentration was fixed at 0.18 mM, 1.80 mM, 5.40 mM and 18.0 mM in this study. Cells were seeded in normal medium as negative control, and cultured in rhBMP-2-containing specific medium as positive control. In subsequent experiments, 0.18 mM- and 18.0 mM-treated rhBMP-2 were named [Ca^2+^]_L_/rhBMP-2 and [Ca^2+^]_H_/rhBMP-2, respectively and were used to explore the interaction of Ca^2+^ and rhBMP-2 in greater detail.

### Interaction of rhBMP-2 and Ca^2+^

Prior to investigate the effect of Ca^2+^ on the rhBMP-2-induced osteoactivity, the interaction between rhBMP-2 and Ca^2+^ was studied. By SPR analysis, it can be observed that over the immobilized rhBMP-2 molecules, Ca^2+^ with concentration ranging from 1.5 × 10^−3^ to 18.0 mM exhibited a rapid binding and attained an equilibrium state within 30 s (Supplementary Fig. S2a). Equilibrium binding data from the highest to lowest Ca^2+^ concentrations yielded average responses of 398.6 to 1.7 relative units that were clearly discernible above the background noise. An equilibrium dissociation constant of 0.023 M was obtained with only one binding site identified.

Next, we investigated the change of the free Ca^2+^concentration of the [Ca^2+^]_L_/rhBMP-2 and [Ca^2+^]_H_/rhBMP-2 in the media without calcium by inductively-coupled plasma atomic emission spectrometry (ICP-AES). The concentrations of free Ca^2+^ reduced with the prolongation of incubation time (Supplementary Fig. S2b). Specifically, the concentration of calcium in [Ca^2+^]_H_/rhBMP-2 was rapidly decreased from 18.0 mM to 2.3 mM.

To further explore the effect of calcium on the C2C12 cells, we studied the change of the free Ca^2+^ concentration inside/outside the C2C12 cells after exposed to the [Ca^2+^]_L_/rhBMP-2 and [Ca^2+^]_H_/rhBMP-2. Obtained from analysis of the extracellular calcium concentration, the results further confirmed that limited concentration of free Ca^2+^ existed in the media, even for the [Ca^2+^]_H_/rhBMP-2 (As shown in Supplementary Fig. S2c). Moreover, the concentrations of intracellular Ca^2+^ were maintained regardless of the concentrations of extracellular Ca^2+^ (Supplementary Fig. S2d). The results demonstrated that calcium could bind with rhBMP-2 and did not lead to obvious alteration of the concentration of free calcium ion inside/outside C2C12 cell.

### Calcium effect on cell morphology

First, we created an *in vitro* environment with various extracellular Ca^2+^ concentrations in specific culture media to evaluate the response of C2C12 cells to Ca^2+^ alone. Concentrations in the range from 0.18 to 18.0 mM of extracellular Ca^2+^ had no obvious effect on cell viability, morphology or ALP activity (Supplementary Fig. S3).

Changes in cell morphology are associated with differentiation^18^,^19^,^20^. Then, we investigated whether different Ca ^2+^ would affect cell shape in the presence of rhBMP-2. As shown in Supplementary Fig. S4a, cells cultured in the presence of 0.18 mM Ca^2+^ had a well spread morphology, suggested high cellular activity^18^. At higher Ca^2+^ concentrations, cells increasingly became elongated and spindle-shaped. After 6 h of culture, cells exposed to 0.18 mM Ca^2+^ were larger area and longer perimeter than those in other groups. These results indicated that changes in cell morphology were induced by exposure to rhBMP-2 combined with low Ca^2+^ concentration.

### Osteogenic differentiation as a function of Ca^2+^ concentration

**Figure 1:**
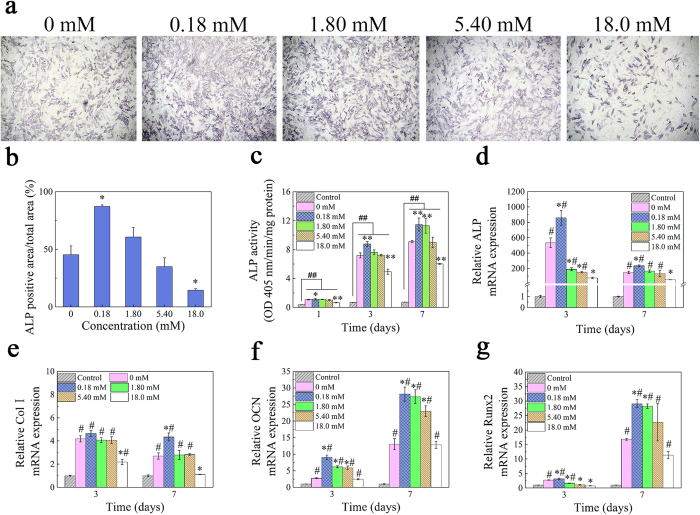
RhBMP-2-induced osteogenic differentiation as a function of calcium ions. (**a**) Alkaline phosphatase activity was stained with BCIP/NBT (20×) at day 3. (**b**) Positive area was quantified by NIH Image J software based on the ALP staining (*p < 0.05, **p < 0.01, compared with free rhBMP-2). (**c**) RhBMP-2 induced ALP activity of C2C12 cells in the presence of Ca^2+^. Cells were cultured with 2.0 μg/mL rhBMP-2 and graded Ca^2+^ concentrations in specific DMEM for 6 h, and then exposed to normal DMEM without rhBMP-2 for another 18 h, 66 h and 162 h, respectively. ALP was measured using soluble substrate p-nitrophenylphosphate. The values represent the mean ± standard deviation (n = 5, ^##^p < 0.01, compared with control (cells cultivated in the DMEM), *p < 0.05, **p < 0.01, compared with free rhBMP-2). (**d–g**) Effects of Ca^2+^ ions on ALP (**d**), Col I(e), OCN (f) and Runx2 (**g**) mRNA expression induced by rhBMP-2. Cells were cultured in rhBMP-2 (2.0 μg/mL) media with various calcium in specific DMEM for 6 h, and then exposed to normal DMEM without rhBMP-2 for another 66 h and 162 h, respectively (n = 3, ^#^p < 0.05, compared with control (cells incubated in DMEM), *p < 0.05, compared with free rhBMP-2).

The effect of Ca^2+^ ions on the rhBMP-2-induced ALP activity was confirmed by an ALP colorimetric assay (Fig. 1a–b) and soluble substrate p-nitrophenylphosphate (PNPP-Na) (Fig. 1c). Figure 1a,b indicated that cells exposed to 0.18 mM Ca^2+^ were induced to differentiate into ALP-positive cells; differentiation decreased relative to control at Ca^2+^ concentrations >5.40 mM (Fig. 1a,b). As shown in Fig. 1c, compared with the cells in the DMEM (control), the ALP activity of cells was significantly enhanced in the rhBMP-2-added media. And a Ca^2+^ concentration-dependent effect on rhBMP-2-induced ALP activity was observed (Fig. 1c). Specifically, cells treated with 0.18 mM Ca^2+^ had higher ALP activity as compared to cells exposed to other Ca^2+^ concentrations and rhBMP-2 only (0 mM, positive control). At early time points (day 1 and 3), ALP activity of cells cultured in 1.80 and 5.40 mM Ca^2+^ was comparable to that without Ca^2+^, while ALP value was higher on day 7 for cells cultured in 1.80 mM Ca^2+^. In contrast, ALP activity was diminished at all time points for cells exposed to 18.0 mM Ca^2+^. Moreover, when cells were incubated in osteogenic media, no significant enhancement was observed with various concentration of calcium compared with control, suggesting that the calcium ions, both in non- osteogenic and osteogenic media, all had little influence on the ALP activity of C2C12 cells.

### Expression of osteogenic gene as a function of Ca^2+^

Osteogenic induction potential was investigated by assessing the expression of the osteogenic genes ALP, collagen I (Col I), osteocalcin (OCN), and runt-related transcription factor 2 (Runx2) on day 3 and 7 by real-time quantitative reverse transcription-polymerase chain reaction (RT-qPCR). ALP and Col I are early and osteocalcin is a late marker for osteoblastic differentiation, while Runx2 is a bone-specific transcription factor that regulates the commitment of pluripotent mesenchymal cells to the osteoblast lineage^21^,^22^.

Compared with control, a statistically significant difference was observed for the osteo-related gene expression in rhBMP-2-containing groups. Comparison of different concentrations of calcium in rhBMP-2-added groups, ALP mRNA expression was markedly higher in cells cultured in 0.18 mM Ca^2+^ but lower for other concentrations relative to free rhBMP-2 on both days (Fig. 1d–g). Similarly, Col I expression was higher at 0.18 mM but lower at 18.0 mM Ca^2+^ on day 7 as compared to positive control (free rhBMP-2), OCN and Runx2 transcript levels were the highest in cells exposed to 0.18 mM Ca^2+^ as compared to the other conditions. These results indicated that the expression of osteogenic genes is stimulated at low Ca^2+^ (0.18 mM) and suppressed at high Ca^2+^ (>1.80 mM) concentrations.

To determine whether BMP-2 and Ca^2+^ act synergistically to induce osteogenesis, the expression levels of osteogenic genes were compared in cells cultured in rhBMP-2 and Ca^2+^ alone or in combination. Compared to free rhBMP-2, 0.18 and 18.0 mM Ca^2+^ alone decreased the expression of ALP, Col I, OCN, and Runx2 after 3 and 7 days (Supplementary Fig. S5). However, ALP, OCN (day 3 and 7), Col I, and Runx2 (day 7) were up-regulated in the presence of rhBMP-2 and 0.18 mM Ca^2+^ but were down-regulated (except for OCN) upon exposure to rhBMP-2 and high (18.0 mM) Ca^2+^ relative to the free rhBMP-2 group on day 3 and 7. These results suggested that a low concentration of Ca^2+^ potentiated the osteogenic induction ability of BMP-2. Compared with rhBMP-2-added groups, calcium alone has little effect on the osteogenic gene expression of C2C12 cells.

### RhBMP-2 localization and receptor binding

**Figure 2:**
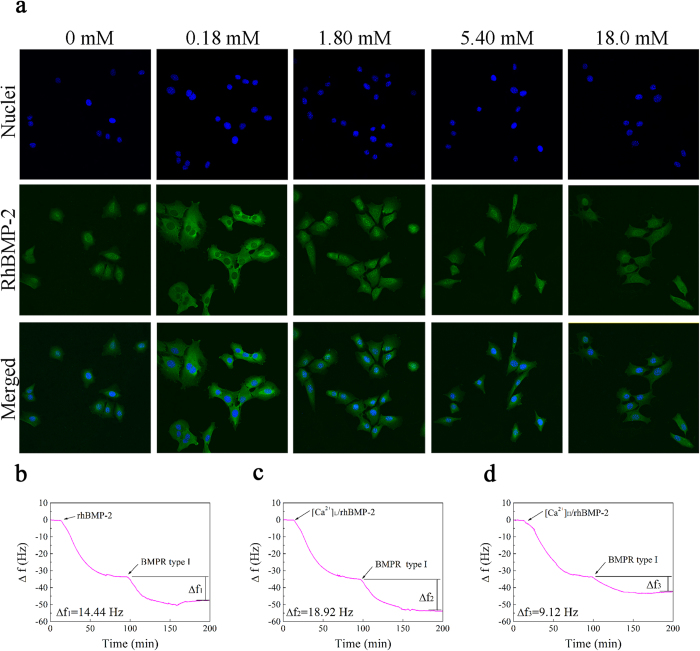
Effect of calcium ions on the binding of rhBMP-2 to cell membranes and BMPR receptors. (**a**) Localization of rhBMP-2 on the cell membranes. C2C12 cells were treated with elevated [Ca^2+^] in the presence of 2.0 μg/mL rhBMP-2 for 6 h. Then, rhBMP-2 was detected with anti-BMP-2 antibody and FITC labeled anti-mouse IgG (green) and cell nuclei were stained with DAPI (blue) for cell orientation (400×). (**b**–**d**) Quantification of BMPRIA to the rhBMP-2 with graded calcium ions using quartz crystal microbalance measurement. Δf curves of the preadsorption of (**b**) rhBMP-2, (**c**) [Ca^2+^]_L_/rhBMP-2, and (d) [Ca^2+^]_H_/rhBMP-2 and the sequential adsorption of BMPRIA (type I) on the chip sensor.

The first step in Ca^2+^-mediated osteogenic differentiation induced by BMP-2 is the binding of BMP-2 to its receptors^23^. In the present study, the rhBMP-2 localization on the cell membranes was evaluated by immunocytochemistry (Fig. 2a). Under all treatment conditions, cells were positive for rhBMP-2 immunoreactivity after 6 h; however, the signal intensity was increased in the presence of Ca^2+^ in a concentration-dependent manner, with the strongest signal observed for 0.18 mM Ca^2+^, suggesting that Ca^2+^ potentiated the localization of rhBMP-2 to cell membranes. Higher Ca^2+^ concentrations had a negative effect on the binding efficiency of rhBMP-2 to cell membranes.

The binding capacity of rhBMP-2 to its receptors was further investigated by quartz crystal microbalance (QCM) technique and typical QCM shifts in frequency ∆f of the adsorption of rhBMP-2, [Ca^2+^]_L_/rhBMP-2 and [Ca^2+^]_H_/rhBMP-2 and corresponding BMPRIA (type I) were shown in Fig. 2b–d. The baseline at the beginning of the adsorption process corresponded to the baseline signal from pure PBS prior to protein injection. After the initial injection of rhBMP-2 with elevated calcium concentration, an immediate decrease in frequency was observed and ∆f was comparable with the value around 33.51 to 34.58 Hz. However, after a subsequent BMPRIA injection, The changes of frequency which are proportional to the mass changes were 14.44 Hz, 18.92 Hz and 9.12 Hz in rhBMP-2, [Ca^2+^]_L_/rhBMP-2 and [Ca^2+^]_H_/rhBMP-2, respectively. The results indicated that the binding efficiency of rhBMP-2 to BMPRIA was influenced by the presence of calcium ions. However, the binding availability of other BMPRs (BMPR-IB and BMPR-II) varied slightly for the [Ca^2+^]_L_/rhBMP-2 and [Ca^2+^]_H_/rhBMP-2 (Supplementary Table S1).

### Signaling pathway analysis

**Figure 3:**
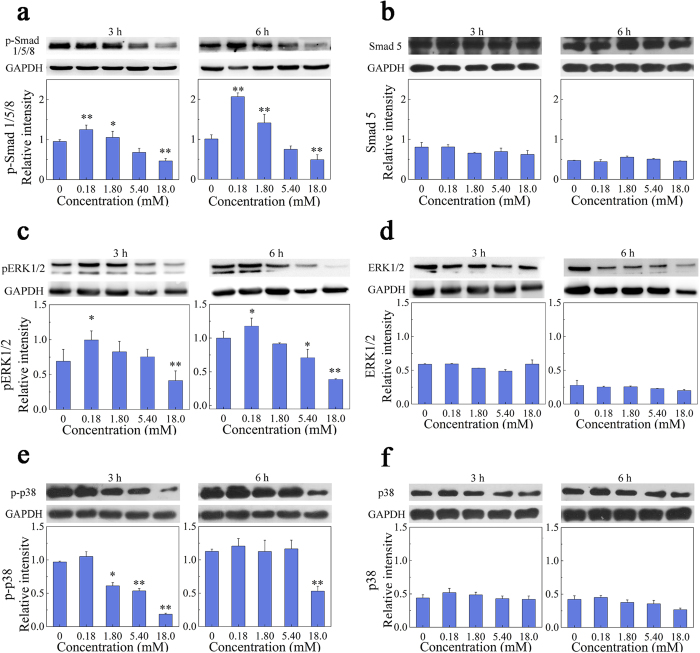
Western blot analysis. Calcium-dependent effects of rhBMP-2 on the phosphorylated Smad 1/5/8 (**a**), total Smad 5 (**b**), phosph-ERK1/2 (**c**), total ERK1/2 (**d**), phosph-p38 (**e**) and total p38 (**f**) were investigated in C2C12 cells exposure to 2.0 μg/mL rhBMP-2 in 2% FBS-containing specific DMEM at 3 and 6 h. Upper panel showed representative electrophoresis images visualized with ECL plus reagents. Bar charts at lower panel indicated band intensity for each of signaling protein at each culture period normalized to GAPDH (n = 3, *p < 0.05, **p < 0.01, compared with free rhBMP-2).

Smad 1, 5 and 8 (Smad 1/5/8) are effectors of the canonical BMP-2 signaling pathway^24^. To determine whether Ca^2+^ induces the phosphorylation of these factors via BMP-2, cells treated with Ca^2+^ and rhBMP-2 were analyzed by western blotting. Smad 1/5/8 phosphorylation was increased at the low concentration of Ca^2+^ (0.18 mM) relative to the positive control (free rhBMP-2). Conversely, phospho-Smad levels were decreased when the concentration of Ca^2+^ was higher than 5.40 mM (Fig. 3a), although the total Smad 5 protein level was unchanged (Fig. 3b). Similarly, an increase in Ca^2+^ concentration from 0.18 to 18.0 mM led to a reduction in ERK1/2 and p38 mitogen-associated protein kinase (MAPK) phosphorylation, while the total ERK1/2 and p38 protein level remained the same (Fig. 3c–f). Therefore, these results indicated the simultaneous involvement of Smad and MAPK (ERK1/2 and p38) signal pathway in the regulation of rhBMP-2-induced osteogenic differentiation of C2C12 cells with various concentrations of Ca^2+^ ions. To clarify the relationship between the Smad and ERK pathway and Ca^2+^ influx via Ca^2+^ channels, cells were pretreated with nifedipine, an inhibitor of L-type Ca^2+^ channels and then stimulated with rhBMP-2/Ca^2+^ to evaluate the levels of Smad/ERK phosphorylation (Supplementary Fig. S6). The results showed that rhBMP-2-induced Smad and ERK phosphorylation was not obviously regulated in the presence of nifedipine, indicating that the Smad/ERK signaling triggered by rhBMP-2 in the presence of calcium would operate independently of Ca^2+^ channels.

### Ca^2+^-induced conformational changes in rhBMP-2

**Figure 4:**
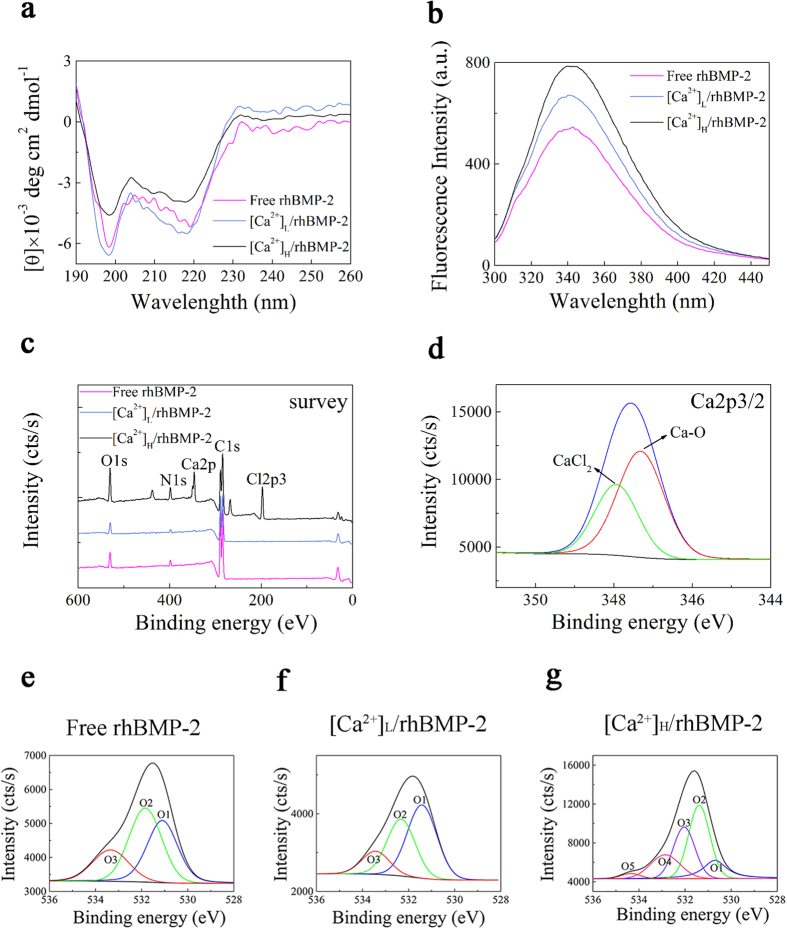
(**a**) Far-UV circular dichroism spectra and (**b**) steady-state fluorescence spectra of the rhBMP-2 in the presence of Ca^2+^ ions. Secondary and tertiary structures of rhBMP-2 were analyzed in the presence of different concentrations of calcium ions. (**c**) XPS survey spectra of C1s, N1s, O1s and Ca2p signals of rhBMP-2 with different [Ca^2+^]. (**d**) High-resolution XPS spectra in the Ca2p2/3 BE region of [Ca^2+^]_H_/rhBMP-2. The actual data were fit with 2 peaks located at the indicated BEs. Blue line represented experimental spectrum, and red/green line represented peak decomposition. (**e–g**) High-resolution XPS spectra in the O1s BE region for 3 samples: free rhBMP-2 (**e**), [Ca^2+^]_L_/rhBMP-2 (**f**) and [Ca^2+^]_H_/rhBMP-2 (**g**). The actual data were fit with 3 peaks in free rhBMP-2 and [Ca^2+^]_L_/rhBMP-2 groups, while the spectrum was dissolved to 5 peaks in [Ca^2+^]_H_/rhBMP-2 group.

The secondary structure of rhBMP-2 in the presence or absence of Ca^2+^ was determined by far-UV CD spectra (Fig. 4a). The secondary structural elements in rhBMP-2, [Ca^2+^]_L_/rhBMP-2, and [Ca^2+^]_H_/rhBMP-2 were summarized in Table 1. For free rhBMP-2 in PBS, the percentages of α-helix, β-sheet and -turn, and random coil were 10.49%, 17.86%, 17.03%, and 54.62%, respectively. In [Ca^2+^]_L_/rhBMP-2, the percentages of β-sheet and -turn were increased by 2.02% and 0.48%, respectively, whereas α-helix and random coil content were decreased by 0.81% and 1.79%, respectively. For [Ca^2+^]_H_/rhBMP-2, the percentages of β-sheet and -turn of rhBMP-2 molecule were up-regulated by 9.56% and 6.92%, while the content of α-helix and random coil decreased by 0.11% and 16.37%, respectively.

The tertiary structure of rhBMP-2 was assessed by steady-state fluorescence spectroscopy. Tryptophan fluorescence emission maxima for free rhBMP-2, [Ca^2+^]_L_/rhBMP-2, and [Ca^2+^]_H_/rhBMP-2 were 343, 342, and 341 nm, respectively (Fig. 4b). Compared to free rhBMP-2, the fluorescence intensities of [Ca^2+^]_L_/rhBMP-2 and [Ca^2+^]_H_/rhBMP-2 were increased by 22.67% and 40.31%, respectively (Table 2).

### X-ray photoelectron spectroscopy

X-ray photoelectron spectroscopy was used to determine changes in the core level binding energy and valence electron structure of rhBMP-2 (Fig. 4c–g). The average C1s, O1s, N1s, and Ca2p binding energies for rhBMP-2, [Ca^2+^]_L_/rhBMP-2, and [Ca^2+^]_H_/rhBMP-2 were determined (Table 3) based on the survey of spectra (Fig. 4c). C1s at 284.8 eV was used as the standard. The appearance of an N signal in the spectra (N1s at 400 eV) provided evidence of rhBMP-2 on the PVDF membranes. The average binding energies of N1s and O1s in [Ca^2+^]_L_/rhBMP-2 and [Ca^2+^]_H_/rhBMP-2 were increased relative to those in free rhBMP-2. Meanwhile, the average binding energy of Ca2p in [Ca^2+^]_H_/rhBMP-2 was decreased from 347.8 eV to 347.6 eV compared to that of pure CaCl_2_^25^. An increase in the binding energies of N1s and O1s in rhBMP-2 and decrease in Ca2p indicated the formation of a Ca^2+^-rhBMP-2 complex in [Ca^2+^]_H_/rhBMP-2^26^. The negligible presence of Ca^2+^ in [Ca^2+^]_L_/rhBMP-2 may be attributable to the low Ca^2+^ concentration.

The electron densities of O and N atoms in the Ca-rhBMP-2 complex were decreased since they were electron pair donors. However, the electron density of Ca^2+^, electron pair acceptor, in the Ca-rhBMP-2 complex was increased relative to that of pure Ca^2+^, indicating the formation of a coordinate covalent Ca-O bond in Ca-rhBMP-2 *via* an interaction between Ca^2+^ and rhBMP-2.

The contributions of particular bonds of rhBMP-2 with O were characterized by quantitative evaluation of O1s. The high-resolution spectra of O1s in rhBMP-2 and [Ca^2+^]_L_/rhBMP-2 were resolved into three components with peaks at 533, 532, and 531 eV, corresponding to O=C, O–C, and O–H bonds, respectively. However, in the case of [Ca^2+^]_H_/rhBMP-2, two peaks appeared, which could arise from the O of carboxylate groups interacting with Ca^2+^ (Fig. 4e–g).

The Ca 2p3/2 core level spectrum for [Ca^2+^]_H_/rhBMP-2 was composed of two peaks: one at 347.8 eV representing CaCl_2_^25^ and one at 347.3 eV representing Ca-O bonds^27^ from the interaction between calcium ions and carboxylate group of rhBMP-2 (Fig. 4d). These results confirmed that the Ca-rhBMP-2 complex was formed by the interaction between Ca^2+^ and the O of the carboxylate group in rhBMP-2.

### Ca^2+^-dependent ectopic bone formation induced by rhBMP-2

The Ca^2+^-dependent induction of osteogenesis by rhBMP-2 was further confirmed by the formation of ectopic bone in the thigh muscle pouches of mice. In the study, we added rhBMP-2 and various CaCl_2_ on FDA-approved gelatin sponge. The rhBMP-2 dosage was based on the reports of our group and the ratio of rhBMP-2 and Ca^2+^ was kept the same as that *in vitro* cell culture. Prior to *in vivo* experiment, we investigated the release of Ca^2+^ and the effect Ca^2+^ on the rhBMP-2-induced osteoactivity from/on the matrix (Supplementary Fig. S7). Release kinetic of calcium from the matrix indicated that the calcium was rapidly released from sponges in the initial 8 h (Supplementary Fig. S7a). But, after addition of rhBMP-2, the release of calcium was greatly inhibited. The result of ALP showed a similar trend with that obtained in 2D culture, confirming again that Ca regulated the rhBMP-2-induced osteogenic differentiation (Supplementary Fig. S7b).

**Figure 5:**
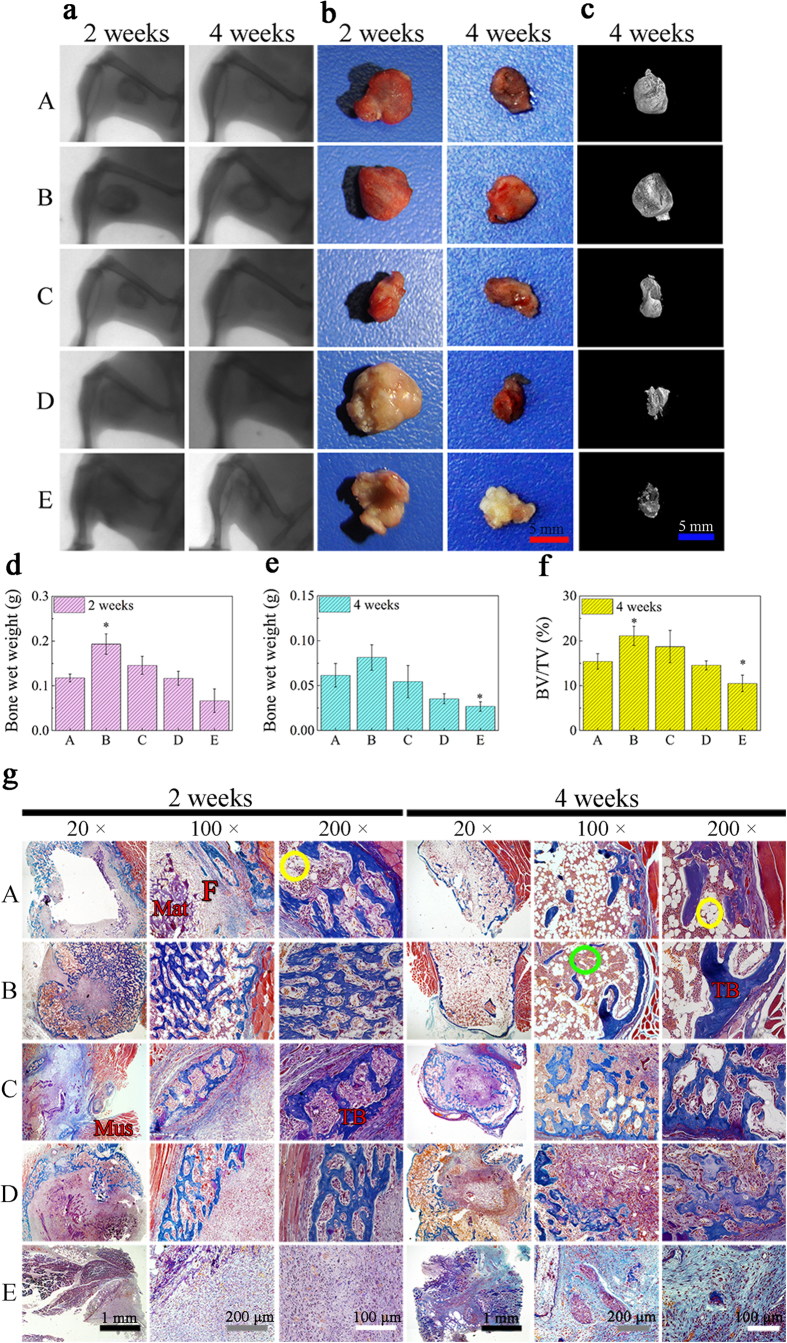
Ectopic bone formation induced by rhBMP-2 with elevated [Ca^2+^] *in vivo*. 5 groups were implanted into mice to induce ectopic bone formation: Group A, free 10 μg rhBMP-2/gelatin sponge; Group B, 0.1 mg CaCl_2_/10 μg rhBMP-2/gelatin sponge; Group C, 1.0 mg CaCl_2_/10 μg rhBMP-2/gelatin sponge; Group D, 3.0 mg CaCl_2_/10 μg rhBMP-2/gelatin sponge; Group E, 10.0 mg CaCl_2_/10 μg rhBMP-2/gelatin sponge. After 2 weeks and 4 weeks, the implants were retrieved and photos were taken by X-ray images (**a**) and digital photos (**b**). Red scale bar is 5 mm. (**c**) 3D μCT reconstructed images of ectopic bone at week 4. Blue scale bar is 5 mm. (**d–e**) Wet weight of ectopic bone at 2 weeks (**d**) and 4 weeks (**e**) (n = 3, *p < 0.05, compared with group A). (**f**) The percentage of bone volume (BV) to total volume (TV) at 4 weeks by μCT. (**g**) Histological evaluation of ectopicly formed bone sections stained with masson/trichrome at 2 weeksand 4 weeks (F: fibrous tissue, Mus: muscle, Mat: material, TB: trabecular bone, yellow circle: medulla ossium flava, green circle: medulla ossium rubra). Black scale bar is 1 mm, grey scale bar is 200 μm and white scale bar is 100 μm.

The X-ray images revealed bone formation in the muscle in mice of Groups A, B and C, visible as an opaque area around the site of implantation. The digital images confirmed the formation of bone in Groups A to C at 2 weeks and in Groups A to D at 4 weeks. The implantation of a 10 μg rhBMP-2/gelatin sponge with or without 0.1 mg CaCl_2_ stimulated new bone formation. There was no bone formation in Group E animals (Fig. 5a,b). Based on the harvested implants (Fig. 5d,e), bone wet weight in the Group B (rhBMP-2/0.1 mg CaCl_2_ implanted) was higher than Group A (rhBMP-2 only) at 2 weeks. At 4 weeks, bone wet weights were significantly reduced in Group E, compared with Group A (p < 0.05). Moreover, Micro-CT images allowed the bone tissues to be distinguished from other tissues in the ectopic bone (Fig. 5c,f). At 4 weeks, the Group B showed a higher content of bone tissues than other groups. However, reduced bone volume/total volume was observed in Group E.

In the free rhBMP-2 group, ectopic bone pellets were filled with fibrous (F, purple area) and some bone tissue (TB, blue area) at 2 weeks, and contained bone marrow (yellow and green circle) and some bone tissue at 4 weeks. With the addition of 0.1 mg CaCl_2_, not only bone marrow but large amounts of trabecular bone and lipocytes (also known as medulla ossium flava, yellow circle, white cells) were observed in the pellets (Fig. 5g). At 4 weeks, the pellets mainly contained lamellar bone within the rich medulla ossium rubra (green circle, red cells). With the addition of 1.0 or 3.0 mg CaCl_2_, fibrous and bone tissue were all observed at each time point. However, there was no ectopic bone formation in mice with the addition of 10.0 mg CaCl_2_. These results indicated that adding low concentrations of Ca^2+^ promoted bone formation, while high concentrations had adverse effects.

## Discussion

Efficient utilization of BMP-2 and avoidance of supraphysiological concentrations remain challenges in the area of regenerative medicine in part because of changes in secondary/tertiary structure and consequent denaturation of BMP-2 during the application *in vitro* and *in vivo*. The effects of the delivery systems, and the environmental factors on the bioactivity of rhBMP-2 have been described previously^5^,^8^,^9^,^10^,^28^. Here, we have shown that calcium ion, a ubiquitous cation in bone repair biomaterials and in the physiological environment, could induce formation of β-sheet/-turn structure and thus modulate the recognition of BMP receptors and osteogenic activity of rhBMP-2.

Data obtained here indicated that Ca^2+^ ion could bind rhBMP-2 rapidly. Even at 18.0 mM, the concentration of rhBMP-2 decreased to 2.3 mM in 30 s and had no obvious effect on the Ca^2+^ concentration inside and outside of cells. Inconsistent with the previous report^29^, Ca^2+^ ions alone even at higher concentrations had little effect on cell viability, cell morphology and osteogenic differentiation both in normal culture media and standard osteogenic medium (Supplementary Fig. S3). These results excluded the possibility that Ca^2+^ ions itself directly mediated osteodifferentiation of C2C12. Together with the fact that BMP-2 can differentiate C2C12 into osteoblast cells, it can be confirmed that the effect of Ca^2+^ on the osteodifferentiation of C2C12 is *via* mediating the rhBMP-2-induced osteogenesis. The ALP activity and expressions of the osteogenic genes ALP, Col I, OCN, and Runx2, along with the formation of ectopic bone *in vivo* all indicated that Ca^2+^ exerted a typical hormetic effect on rhBMP-2-induced osteogenic differentiation: an enhancement at low Ca^2+^ concentrations (0.18 mM) and an inhibition at high concentrations (especially > 5.40 mM).

As a member of the TGF-β superfamily, BMP-2 binds to BMPR-IA/IB with high affinity, and subsequently recruits the lower affinity BMPR-II to form a signaling complex, which in turn initiates a downstream signal transduction cascade (Smad and MAPK signaling pathways)^30^,^31^,^32^,^33^,^34^,^35^. Therefore, the role of Ca^2+^ in the regulation of BMP-2-induced osteogenic differentiation requires an understanding of BMP-2 binding to its receptors and expression levels of associated signaling pathways at various Ca^2+^ concentrations. The results obtained here indicated that not only the binding efficiency of rhBMP-2 to cell membranes, but also the binding capacity to BMPR-IA/IB with high affinity were increased by a low concentration of Ca^2+^, but were inhibited at a high concentration. High Ca^2+^ also decreased the levels of phosphorylated Smad 1/5/8, ERK1/2 and p38. Ca^2+^, a ligand for several G-protein coupled receptors (GPCRs), can enter the cell via ion channels such as voltage-gated Ca^2+^ channels (VGCCs), acid sensing ion channels (ASIC), etc, and thus results in phosphorylation of extracellular signal pathway^29^. L-type VGCCs (L-VGCCs) could often be activated by CaP crystals or the dissolved Ca^2+^ and thus led to an increase in ALP expression. Here, Nifedipine, a L-VGCC blocker^36^, was found almost no effect on the osteogenesis of C2C12, confirming that Ca^2+^ exerted effect via binding to rhBMP-2 and rhBMP-2-mediated signaling pathway, but not a negative feedback loop through the calcium receptors (Supplementary Fig. S6). In light of these findings, it can therefore be concluded that Smad and MAPK (ERK1/2 and p38) signaling pathways play critical role in Ca^2+^-mediated alteration of osteogenic differentiation of rhBMP-2. Morphological changes may be an additional factor that influences osteogenic differentiation (Supplementary Fig. S4)^29^.

Some cations, such as La^3+^, Ca^2+^, and Mg^2+^ often mediate activity of proteins via alteration of the secondary structure^37^,^38^. As anticipated, the CD spectra indicated that Ca^2+^ ions had little effect on α-helix, but induced the formation of β-turn/-sheet, especially at high concentrations. Similar phenomena have previously been observed by Nagano, A, where the β-sheet of silk protein was promoted by the binding of Ca^2+^ or Zn^2+^ with proteins^39^,^40^.The steady-state fluorescence spectroscopic analysis showed a clear blue shift and enhanced fluorescence intensity at high Ca^2+^ concentrations, indicating an improvement of the hydrophobic environment for tryptophan residues, which is related to protein aggregation and conformational change. These results confirm that high concentration Ca^2+^ leads to increased order within aggregation of BMP-2 molecules.

**Figure 6:**
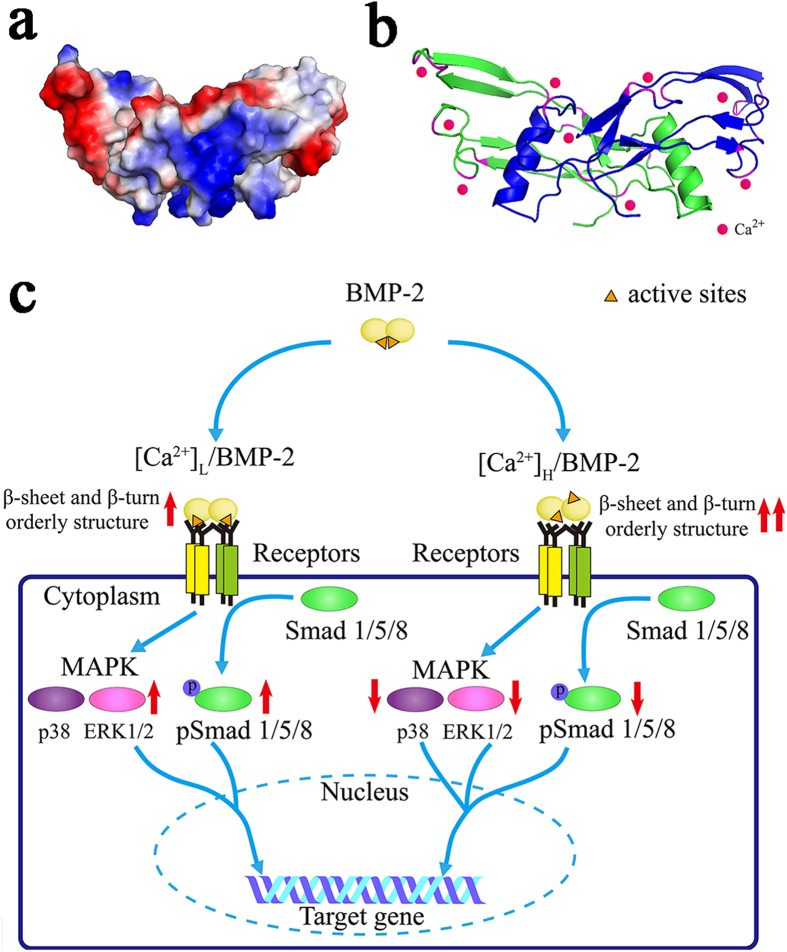
Schematic depiction of the effect of Ca^2+^ on the conformation and bioactivity of rhBMP-2. (**a**) Charge distribution on the surface of rhBMP-2. Blue color represented the positive charge, red color refered to the negative charge and white color represented hydrophobic. (**b**) Band display of rhBMP-2. α-helices were indicated as spiral, β-strands as arrow. The subunits were color-coded blue and green, respectively. The amino acid residues of negative charge represented for magenta color. Calcium ions were color-coded pink. (**c**) Effect of Ca^2+^ on the rhBMP-2-induced osteogenic differentiation. Ca^2+^ ion at low concentration facilitated for the binding capacity of BMP-2 with its receptors on cell membrane and thus enhanced Smad1/5/8, and MAPK signaling transduction, which further stimulated expression of osteogenic marker genes and the ALP activity. Additionally, increasing Ca^2+^ attenuated the binding capacity to BMPR-IA, down-regulated the signaling transductions of Smad1/5/8, MAP Kinases and consequently reduced the rhBMP-2-induced gene expression and ALP activity.

It is reported that Ca^2+^ interacts with proteins through negatively charged carboxylate ions of Glu and Asp^41^,^42^,^43^,^44^,^45^. And 3–4 acidic residues in osteopontin are often needed to chelate divalent cations. It is possible that water molecules, carbonyl groups of the protein backbone, or other amino acid side chains form coordinating bonds^45^. As a homodimer linked with inter-chain disulfide bridges, the images of the charge distribution in the surface and band structure of the BMP-2 molecule, which are calculated using the Electrostatic Potential module in the software of Pymol, are shown in Fig. 6a,b. BMP-2 molecule contains 12 Asp, 10 Glu, and 2 terminal carboxyl residues. That is to say, there are 24 carboxylate groups in the BMP-2 might register with Ca^2+^ and in turn mediate the conformation of BMP-2. The structural analyses provide evidence for the coordination of O in the carboxylate group of the rhBMP-2 molecule with Ca^2+^, resulting in the formation of a Ca-rhBMP-2 in the presence of high concentration of Ca^2+^. Cation/protein complexes have been proven to facilitate the formation of inter- or intramolecular hydrogen bonds within proteins and thereby cause an amino acid chain to fold into β-turn/-sheet^40^,^46^,^47^. With these reports and the data obtained here, it can be inferred that formation of a Ca-rhBMP-2 complex may be responsible for the increase in the ordered structure of rhBMP-2 at high Ca^2+^ concentrations. However, given our findings, the site specific binding information between calcium and BMP-2 is unclear.

The data obtained and discussed above led us to a proposed schematic illustration for the effect of Ca^2+^ on BMP-2-induced osteogenic differentiation and was shown in Fig. 6c. At low Ca^2+^ concentrations, a weak interaction between Ca^2+^ and rhBMP-2 leads to a slight increase in β-sheet/-turn content and a relaxation of the rhBMP-2 molecule, which increases the exposure of active sites in the protein^26^ and thereby enhances its bioactivity. While an increase in Ca^2+^ concentration leads to the formation of a Ca-BMP-2 complex followed by a marked increase in the β-sheet/-turn content of the BMP-2 molecule. As a result, the binding of BMP-2 to its receptor and the activation Smad1/5/8 and MAPK signaling pathways, such as ERK1/2, p38, are inhibited, which attenuates the capacity for rhBMP-2 to induce osteogenic differentiation.

Several factors including pH, delivery molecules, and release mode among others have been shown to exert effect on the BMP-2-induced osteogenesis bioactivity^28^,^48^. The present study for the first time demonstrated that the extracellular Ca^2+^ level can also modulate the conformation of BMP-2 and thus the capacity for inducing osteogenic differentiation. This concept, therefore, has significant implications in understanding the interactions of cation ion and BMP-2 and assisting in the design and fabrication of BMP-2-based scaffold for bone regeneration.

## Methods

### Alkaline phosphatase (ALP) activity assay

A two-stage cell culture process was used to detect ALP activity. C2C12 was first cultured in rhBMP-2-containing specific DMEM for 6 h and followed by the normal DMEM without rhBMP-2 for another 18 h, 66 h and 162 h. After that, cells were lysed in 50 μL of 1% Nonidet P-40 (NP-40) solution and mixed with 100 μL of 1 mg/mL p-nitrophenylphosphate (PNPP-Na) solution. After 15 min incubation at 37 ^o^C, the reaction was ended by adding 50 μL of 0.1 M NaOH. The optical density (OD) was measured at the wavelength of 405 nm using an enzyme-linked immunoadsorbent assay plate reader (SPECTRAmax 384, Molecular Devices, USA). The total protein content was quantified by the bicinchoninic acid protein assay kit (Beyotime, Shanghai, China). The ALP activity was expressed as OD values divided by the reaction time and total protein quantity (n = 5).

ALP stain assay was also employed to determine the ALP activity histochemically. After fixation of C2C12 cells in 2.5% glutaraldehyde for 10 min on ice, cells were incubated in alkaline phosphatase stain solution (Beyotime, Shanghai, China) for 30 min at room temperature. Stained cells were visualized with inverted light microscope (TE2000U, Nikon Corp., Japan) and quantified using NIH Image J software (NIH, Bethesda, MD, USA).

### Gene expression analysis

Total RNA was extracted using Trizol Reagent (Takara, Tokyo, Japan) and concentration was determined by measuring optical absorbance at 260 nm. First strand complementary DNA (cDNA) was synthesized from 2 μg RNA using PrimeScript RT reagent Kit (Takara, Tokyo, Japan). ALP, Collagen type I (Col I), osteocalcin (OCN) and runt-related transcription factor 2 (Runx2) were evaluated, while GAPDH gene was used as the house keeping gene. All experiments were performed in triplicate. The forward and reverse primer sequences were listed in Supplementary Table S2.

### Binding capacity to cell membranes of rhBMP-2

The binding efficiency of rhBMP-2 to cell membranes was assessed using the immunofluorescence assay. C2C12 cells were seeded as mentioned before. Briefly, the cells were fixed with 2.5% glutaraldehyde for 15 min at 4 ^o^C and nonspecific binding sites were blocked with 3% FBS/PBS solution for 1 h at 37 ^o^C. Afterwards, cells were incubated with anti-BMP-2 antibody (R&D systems Inc., Minneapolis, USA) and a dilution of FITC-conjugated goat-anti-mouse IgG (Sigma, St. Louis, USA) to stain BMP-2. Cell nucleus were stained with DAPI solution for 15 min at RT. RhBMP-2 and cell nucleus were measured using confocal microscope at the wavelength of 488 nm and 405 nm, respectively.

### Quartz Crystal Microbalance (QCM) measurements

The QCM (E1, Q-Sense AB) measurements were performed at 25.0 ± 0.05 ^o^C by real time *in situ* monitoring of Δf at 25 MHz in the QCM cell chamber. After PBS filling by an injection of 1.0 mL, the Δf curves were completely stabilized for 10–20 min. The adsorption was measured after the exchange with 2.0 mL of 50 μg/mL rhBMP-2 or mixed solution of rhBMP-2/Ca^2+^ dispersed in PBS to stabilize the Δf curves for 80–100 min. The adsorption of the rhBMP-2 receptors after the exchange with 10 μg/mL BMPRIA, BMPRIB was monitored for 100 min.

### Signaling pathway analysis

Western blot assay was employed to analyze signaling pathway of C2C12 cells triggered by rhBMP-2 with elevated [Ca^2+^]. The cells were extracted by RIPA lysis buffer. Equal amount of proteins (50 μg) were then subjected to 8% SDS-polyacrylamide gel electrophoresis (PAGE) and then electro-transferred to a polyvinylidene difluoride membrane (PVDF, Bio-Rad, USA). The membranes were blocked by 3% BSA at room temperature for 60 min and incubated with appropriate primary antibodies at 4 ^o^C overnight. The primary antibodies used for analysis were anti-phospho-Smad 1, 5, 8, and anti-Smad 5, anti-phospho-ERK1/2-MAPK, anti-ERK1/2-MAPK, anti-phospho-p38-MAPK, anti-p38-MAPK (Cell Signaling Technology, Beverly, MA, USA) and anti-GAPDH (Sigma-Aldrich, Saint Louis, MO, USA). After 90 min incubation with horseradish peroxidase (HRP)-conjugated goat anti-rabbit and rabbit anti-mouse (Beyotime, China), the membranes were visualized using the ECL plus reagents (Amersham Pharmacia Biotech, USA) by Image Quant LAS 4000 (GE, USA).

### Far-ultraviolet circular dichroism spectroscopy

The secondary structures of rhBMP-2 affected by calcium ions were detected by far-UV circular dichroism spectroscopy (Model J-715, Tokyo, Japan). Far-UV CD spectra were acquired at the wavelength of 190–260 nm in quartz cell with 0.1 cm path length and a time constant of 0.25 s at a scan rate of 100 nm/min. Three such runs were averaged, calculated net of buffer, and smoothed using the accompanying software program. CDNN Deconvolution software (version 2.1) was employed for estimation of secondary structure content.

### Steady-state fluorescence spectroscopy

Steady-state fluorescence spectroscopy measurement was recorded with a spectrofluorometer (RF-5301, Shimadzu, Japan) at 25 ^o^C in a thermostated cuvette holder. The measurements were performed in a 1 cm × 1 cm quartz cuvette with 3 mL of sample. The intrinsic protein fluorescence was monitored between 300 and 450 nm with an excitation wavelength of 281 nm. The spectra were recorded with 0.1 s integration time per 1 nm increment. The excitation and emission slits were set to 0.5 mm and 0.5 mm separately.

### X-ray photoelectron spectroscopy (XPS) spectra

XPS spectra were carried out on an ESCALAB 250Xi X-ray photoelectron spectrophotometer (Thermo Fisher, USA) with an Al Kα source. The specimens were prepared as follow: 1 cm × 1 cm PVDF membranes were rinsed with water and immediately immersed in the solution of the rhBMP-2 and/or Ca^2+^. After 24 h incubation at 4 ^o^C, the membranes were dried in the vacuum condition overnight.

### Ectopic bone formation

This study was performed in strict accordance with the NIH guidelines for the care and use of laboratory animals (NIH Publication No. 85e23 Rev. 1985) and was approved by the Research Center for Laboratory Animal of Shanghai University of Traditional Chinese Medicine. Ectopic bone formation was evaluated using the mouse hindlimb muscle pocket model. Forty female C57BL/6 mice (eight weeks old, Silaike Inc. Shanghai, China) were randomly allocated into week 2 and 4 time points. At each time point, twenty mice received surgery that the gelatin pellets prepared before were surgically implanted into right leg muscle pouches and randomly assigned to one of the following five groups: Group A, free rhBMP-2/gelatin sponge (n = 4); Group B, 0.1 mg CaCl_2_/rhBMP-2/gelatin sponge (n = 4); Group C, 1.0 mg CaCl_2_/rhBMP-2/gelatin sponge (n = 4); Group D, 3.0 mg CaCl_2_/rhBMP-2/gelatin sponge (n = 4); Group E, 10.0 mg CaCl_2_/rhBMP-2/gelatin sponge (n = 4).

At 2 and 4 weeks after surgery, the mice were scanned using a Kodak multimodel imaging system (Carestream Health Inc., USA) with X-ray to evaluate the ectopic bone formation. Then the mice were sacrificed and the implants were harvested. The weights of all these samples were obtained at wet contains. All procedures were carried out under aseptic conditions. The harvested bone specimens were imaged with μCT (GE Explore Locus SPmicroCT, USA) to determine the new bone volume. The specimens were used for histological evaluation. Briefly, ectopic bone samples were fixed with 4% neutral buffered formalin for 48 h at 4 ^o^C and then decalcified in 12.5% EDTA. Following dehydration in a graded series of alcohol, the specimens were embedded in paraffin and sectioned at 4-mm thickness. Masson/trichrome staining was performed on serial sections and images were taken using inverted light microscope.

### Data analysis

All numerical data were expressed as the mean ± standard deviation. Statistical analysis was performed with one-way analysis of variance (ANOVA). A value of p < 0.05 was considered as statistical significance.

## Additional Information

**How to cite this article**: Zhang, W. *et al.* Calcium ion-induced formation of β-sheet/-turn structure leading to alteration of osteogenic activity of bone morphogenetic protein-2. *Sci. Rep.* **5**, 12694; doi: 10.1038/srep12694 (2015).

## Supplementary Material

Supplementary Information

## Figures and Tables

**Table 1:** Secondary structure content of rhBMP-2 in the presence calcium ions.

Sample	α-helix	β-sheet	β-turn	Random coil
Free rhBMP-2	10.49%	17.86%	17.03%	54.62%
[Ca^2+^]_L_/rhBMP-2	9.68%	19.88%	17.51%	52.83%
[Ca^2+^]_H_/rhBMP-2	10.38%	27.42%	23.95%	38.25%

**Table 2:** Fluorescence parameters of rhBMP-2 w/wo calcium ions.

Sample	λ_em_max (nm)	Fluorescence Intensity (FI)	Changes of FI
Free rhBMP-2	343	548.19	/
[Ca^2+^]_L_/rhBMP-2	342	672.45	22.67%
[Ca^2+^]_H_/rhBMP-2	341	757.32	40.31%

**Table 3:** The average binding energy of C1s, O1s, N1s and Ca2p in free rhBMP-2, [Ca^2+^]_L_/rhBMP-2 and [Ca^2+^]_H_/rhBMP-2.

Sample	C1s/eV	O1s/eV	N1s/eV	Ca2p/eV
Ca^2+^	/	/	/	347.8
Free rhBMP-2	284.8	531.5	399.8	/
[Ca^2+^]_L_/rhBMP-2	284.8	531.8	399.9	/
[Ca^2+^]_H_/rhBMP-2	284.8	532.1	400.0	347.6
