# Author Correction: Calcium ion-induced formation of β-sheet/-turn structure leading to alteration of osteogenic activity of bone morphogenetic protein-2

**DOI:** 10.1038/s41598-021-90711-8

**Published:** 2021-05-19

**Authors:** Wenjing Zhang, Hongyan He, Yu Tian, Qi Gan, Jing Zhang, Yuan Yuan, Changsheng Liu

**Affiliations:** 1 1The State Key Laboratory of Bioreactor Engineering, East China University of Science and Technology, Shanghai, 200237 PR China; 2 Key Laboratory for Ultrafine Materials of Ministry of Education, East China University of Science and Technology, Shanghai, 200237 PR China; 3 Engineering Research Center for Biomedical Materials of Ministry of Education, East China University of Science and Technology, Shanghai, 200237 PR China

Correction to: *Scientific Reports* 10.1038/srep12694, published online 27 July 2015

This Article contains errors.

As a result of a figure assembly error, in Figure 5a panel C/2 weeks is a duplication of panel A/2 weeks. The correct Figure 5 appears below as Figure 1.

**Figure 1:**
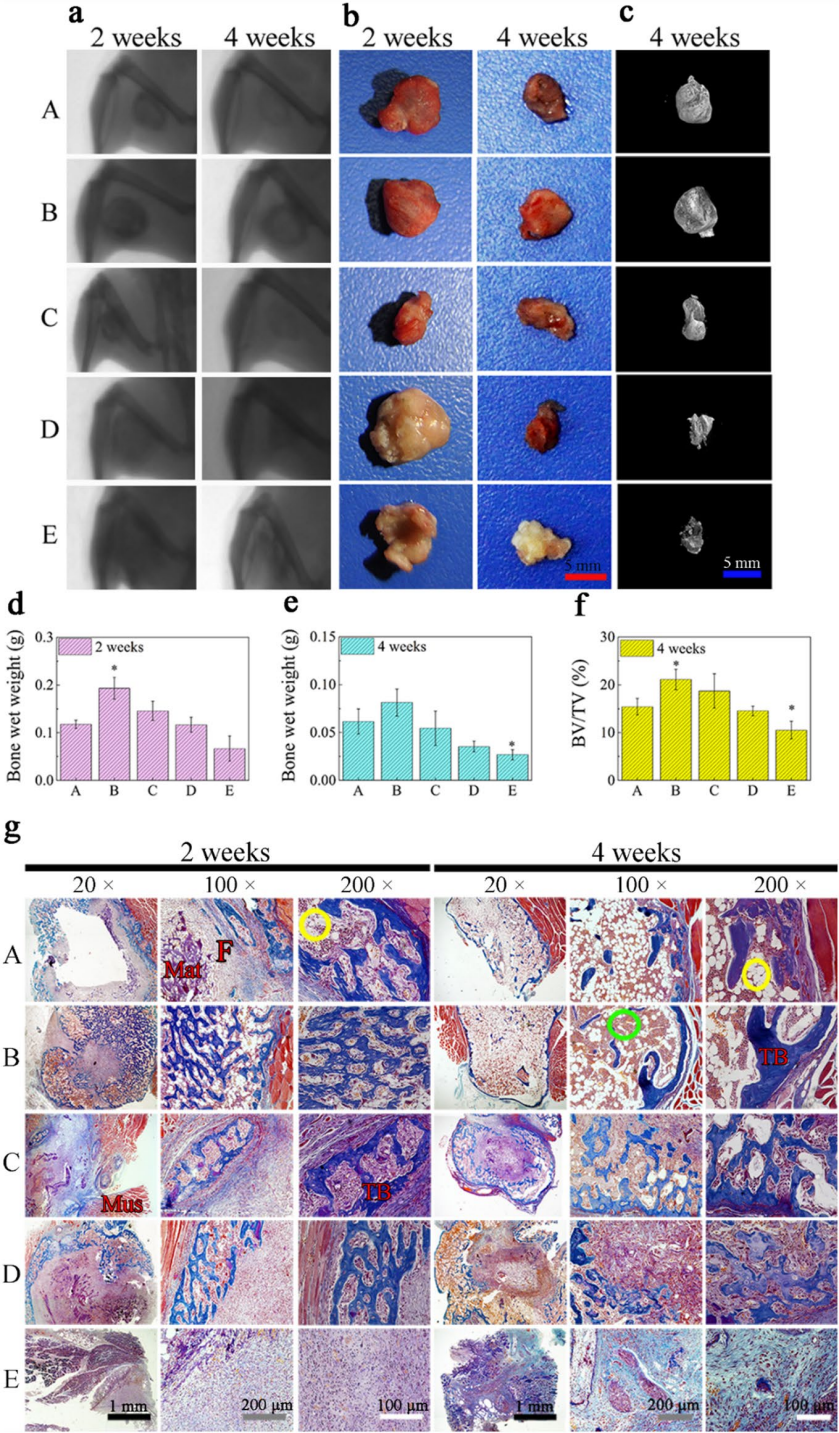
The correct version of Figure 5.

This change does not affect the conclusions of the Article.

