# Supplementary material for: Calcium ion-induced formation of β-sheet/-turn structure leading to alteration of osteogenic activity of bone morphogenetic protein-2
Source: Sci Rep. 2015 Jul 27;5:12694. doi: 10.1038/srep12694 (PMC4515877; doi:10.1038/srep12694)
Supplement: Supplementary Information [file srep12694-s1.pdf]

Supplementary data

Calcium ion-induced formation of β-sheet/-turn structure leading to alteration of osteogenic activity of bone morphogenetic protein-2

Wenjing Zhang[1, 2], Hongyan He[3], Yu Tian[1,2], Qi Gan[2, 3], Jing Zhang[3], Yuan Yuan[1, 3] *, Changsheng Liu[1, 2, 3] *

[1] The State Key Laboratory of Bioreactor Engineering, East China University of Science and Technology, Shanghai 200237, PR China

[2] Key Laboratory for Ultrafine Materials of Ministry of Education, East China University of Science and Technology, Shanghai 200237, PR China

[3]Engineering Research Center for Biomedical Materials of Ministry of Education, East China University of Science and Technology, Shanghai 200237, PR China

Email: liucs@ecust.edu.cn

Materials and methods

Materials. Methyl thiazolyl tetrazolium (MTT), 2-(4-Amidinophenyl)-6-indolecarbamidine dihydrochloride (DAPI), fluorescein isothiocyanate-Phalloidin (FITC-Phalloidin) and $CaCl_2$ were from Sigma-Aldrich (CA, USA). BCA protein assay kit and BCIP/NBT alkaline phosphatase color development kit were purchased from Beyotime Biotech Co. Ltd (Jiangsu, China). Recombinant human bone morphogenetic protein-2 (rhBMP-2, 26 kD) was generously provided by Shanghai Rebone Biomaterials Co. Ltd. (Shanghai, China). Trizol reagent, PrimeScript RT reagent kit and SYBR Premix Ex Taq^{TM} were from Takara (Tokyo, Japan). All reagents used in cell culture were from Gibco (Grand Island, NY).

Cell culture. The mouse myoblast cell line C2C12, with osteoblastic potential, was purchased from the American Type Culture Collection (ATCC). C2C12 cells were cultured in 37.5 cm^2 flasks with Dulbecco's modified Eagle's medium (DMEM, growth medium) containing 10% fetal calf serum, antibiotics (100 U/mL penicillin-G, and 100 mg/mL streptomycin)) at humidified atmosphere of 5% CO_2/95% air until confluence, then detached with 0.25% trypsin/0.03% ethylenediamine tetraacetic acid (EDTA), and the cell density was calculated and used at the desired density in later experiments.

Cell viability. MTT assay was performed to evaluate cell viability cultured in different treatments. 30 μL of 5.0 mg/mL MTT solution was added and incubated at 37 ^oC to form formazan crystals. After 4 h, the formazan solubilized with 100 μL of dimethyl sulfoxide (DMSO). The absorbance of the formazan-DMSO solution was read at 492 nm using an enzyme-linked immunoadsorbent assay plate reader (SPECTRAmax 384, Molecular Devices, USA). Results were reported as the percentage ratio of $OD_{sample}/OD_{control} \times 100\%$ (n=5).

Cell morphology. Cell morphology was determined by fluorescent staining. C2C12 cells were first seeded at a density of 3×10^4/mL and incubated in a series of culture media containing rhBMP-2 and different [Ca^{2+}]. After 6 h incubation, media were refreshed by normal DMEM for further cultivation. Samples were fixed with Formalin solution (3.7% formaldehyde in PBS) for 15 min on ice, subsequently stained by

FITC-Phalloidin for the cell cytoskeleton. DAPI solution was added to stain cell nuclei for 10 min. The specimens were observed using Confocal Laser Scanning Microscope (CLSM, Nikon A1R, Japan). Area and perimeter of isolated cells were analyzed with the grain analysis tool by using Image J software.

Surface plasmon resonance (SPR) analyses. Surface plasmon resonance analyses were performed using BIAcore 2000 optical biosensors equipped with research-grade CM5 sensor chips (GE Healthcare Bio-Sciences, AB, Sweden). RhBMP-2 was immobilized using amine-coupling chemistry on the CM5 sensor chips [1]. Solution of calcium ions was injected over the rhBMP-2 surfaces from 18.0 to 1.5×10^{-3} mM in two fold dilutions at a flow rate of 30 μL/min. To determine equilibrium dissociation constants for the calcium/rhBMP-2 interaction, the equilibrium response data were fit to an independent-binding-sites model [2]:

$$R = \sum_{i} \frac{R_{max}(C/Kd_i)}{1 + C/Kd_i}, \qquad (1)$$

Where R_{max} is the maximal response, C is the calcium concentration, and K_d is the equilibrium dissociation constant. Here, i = 1 for a single-site interaction and i = 2 for multiple binding sites. Fitting was done using nonlinear curve fitting within Origin 8.0.

Measurement of changes of free calcium ion. Free Ca^{2+} concentration was determined by inductively-coupled plasma atomic emission spectrometry (ICP-AES, IRIS Advantage ER/S). The samples was prepared as follows: after the incubation of indicated time (10, 30 and 60 s), the mixture of rhBMP-2 and 0.18 or 18.0 mM Ca^{2+} was concentrated using centrifugal filter device (Merck Milipore, Ireland). The concentration of calcium in the filtrate was analyzed by ICP-AES.

The concentrations of extracellular Ca^{2+} were also assessed by ICP-AES. The cells were cultured 0.18 mM or 18.0 mM Ca^{2+}-containing media with/without rhBMP-2. After 6 h incubation, the media were removed from the plates and centrifuged for removing the protein using centrifugal filter device. The concentrations of calcium in the filtrate were determined using ICP-AES.

Determination of intracellular calcium ion concentration. Cells were exposed to 18.0 mM Ca^{2+} in the

DMEM for 6 h for the determination of the intracellular calcium ion concentration ($[Ca^{2+}]_i$). Cultures grown in 96-well plates were loaded with 4 μM fluo-3 (Beyotime Institute of Biotechnology), which was dissolved in dimethyl sulphoxide (1:1000) in HEPES Hank's salt solution containing bovine serum albumin (1 mg/ml) for 30 min in the dark at 37 °C, and were subsequently washed twice with fresh HEPES Hank's salt solution containing bovine serum albumin, followed by a further incubation for 30 min. Fluorescence was measured at an excitation wavelength of 485 nm and an emission wavelength of 535 nm in microplate reader. The relative fluorescent unit was normalized to the value obtained with the cells cultured in DMEM.

Release kinetic of calcium from the gelatin sponge. The well mixed solutions of 10.0 mg $CaCl_2$ with or without 10 μg rhBMP-2 were blotted onto a gelatin sheet (75 mm × 70 mm × 5 mm) cut from absorbable gelatin sponge (Xiang'en Med, Jiangxi, China) and freeze dried. The sponges were immersed in 200 mL specific media (without calcium). The culture medium were collected at the indicated time (2, 4, 6, 8, 10, 12, 24, 36 and 48 h) and analyzed using ICP-AES for the calcium concentration.

Cell culture on the gelatin sponge with Ca^{2+} and rhBMP-2. The gelatin sponges were prepared as mentioned before. However, based on the fact that the concentration of rhBMP-2 needed in vivo was much higher than that in vitro, the mass of rhBMP-2 (1 mL DMEM) in 3D culture in vitro experiment for mimicking the procedure in vivo was decreased to 2.0 μg, which coincided with 2.0 μg/mL rhBMP-2 in the previous cell trials. Similarly, the mass of $CaCl_2$ was changed to 0.02 mg, 0.2 mg, 0.6 mg and 2.0 mg, respectively. After 24 h incubation in growth media, cells were cultured with sponges in the specific media for 6 h. Then, the sponges were removed from the plates and the culture media were refreshed with normal DMEM for further differentiation.

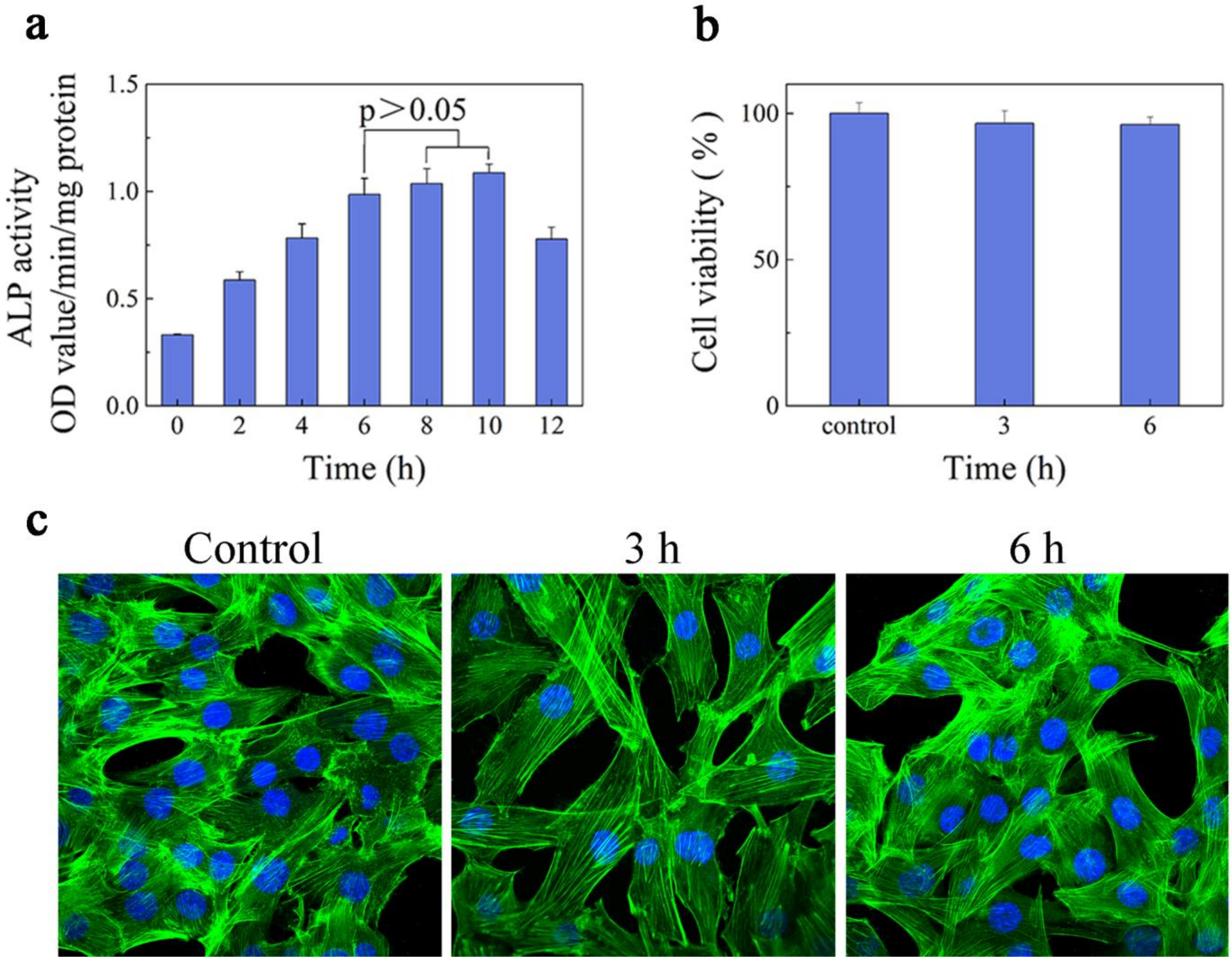

Fig S1 Determination of culture time of C2C12 cells exposed to rhBMP-2-containing specific DMEM medium in the first stage cell culture. (a) ALP activity vs. culture time of C2C12 with rhBMP-2-containing specific DMEM medium. C2C12 cells were first exposed to rhBMP-2-containing specific DMEM medium for various time and thereafter transferred into regular DMEM for another cultivation time (The whole culture time was all kept at 72 h). (b) Cell viability of C2C12 in specific DMEM media without Ca^{2+} and Mg^{2+}. The cells were incubated in specific medium for 3 and 6 h and MTT assay was employed for analyzing the effects of specific media on the cell viability. n=5, $p > 0.05$. (c) Fluorescence images of C2C12 cells cultured in specific medium for 3 and 6 h. The cells were stained by DAPI (blue) for cell nucleus and phaloidin (green) for actin skeleton.

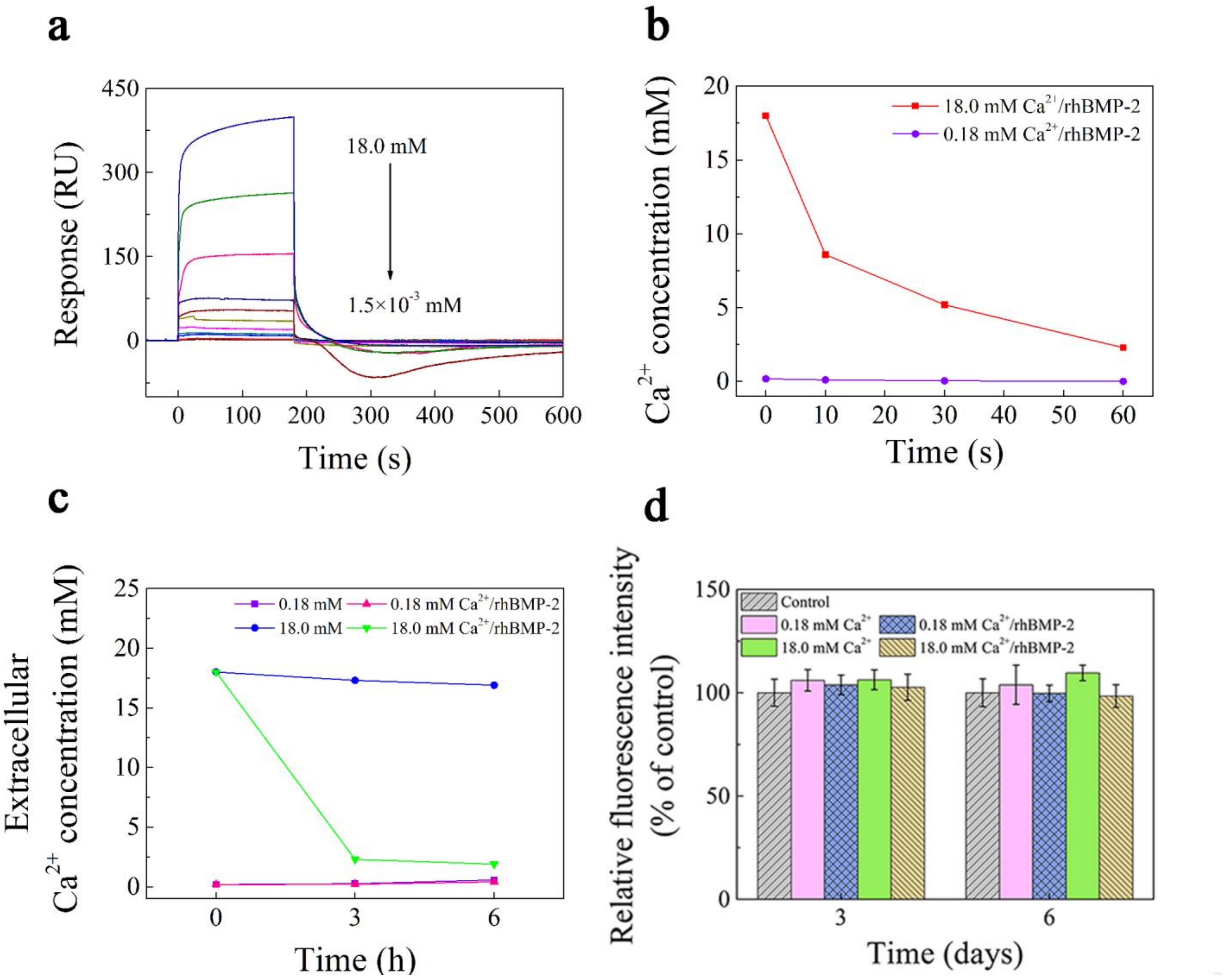

Fig S2 (a) Calcium/rhBMP-2 equilibrium study. Sensorgrams obtained from injections of calcium (18.0–1.5 $\times 10^{-3}$ mM). The fit of the data to the model described by Eq. (1) assuming i=1 for the one-site reaction. (b) Concentration of free Ca^{2+} in the media after binding with rhBMP-2. RhBMP-2 was first mixed with 0.18 mM Ca^{2+} or 18.0 mM Ca^{2+} and incubated for indicated time (10 s, 30 s and 60 s). The mixtures were concentrated using centrifugal filter device and then the Ca^{2+} concentration in the filtrate was analyzed using ICP-AES from the mixture of rhBMP-2 and calcium. The concentration of free calcium in the media was decreased with the prolonging of the incubate time. (c) Alteration of extracellular calcium ion concentration. After 3 h and 6 h cultivated with extracellular Ca^{2+} (0.18 mM and 18.0 mM) with or without rhBMP-2, the concentrations of extracellular Ca^{2+} were analyzed using ICP-AES. (d) Intracellular calcium ion concentration. The intracellular Ca^{2+} concentrations were assessed with fluo-3 assay kit. Briefly, C2C12 cells were exposed to Ca^{2+} (0.18 mM and 18.0 mM) with or without rhBMP-2 for 3 h and 6 h and then were treated with 4 μM fluo-3. Fluorescence was measured at an excitation wavelength of 485 nm and an emission wavelength of 535 nm. The relative fluorescent intensity was normalized to the value obtained with cells cultured in DMEM. The

percentage of fluorescence intensity represented the concentration of intracellular calcium.

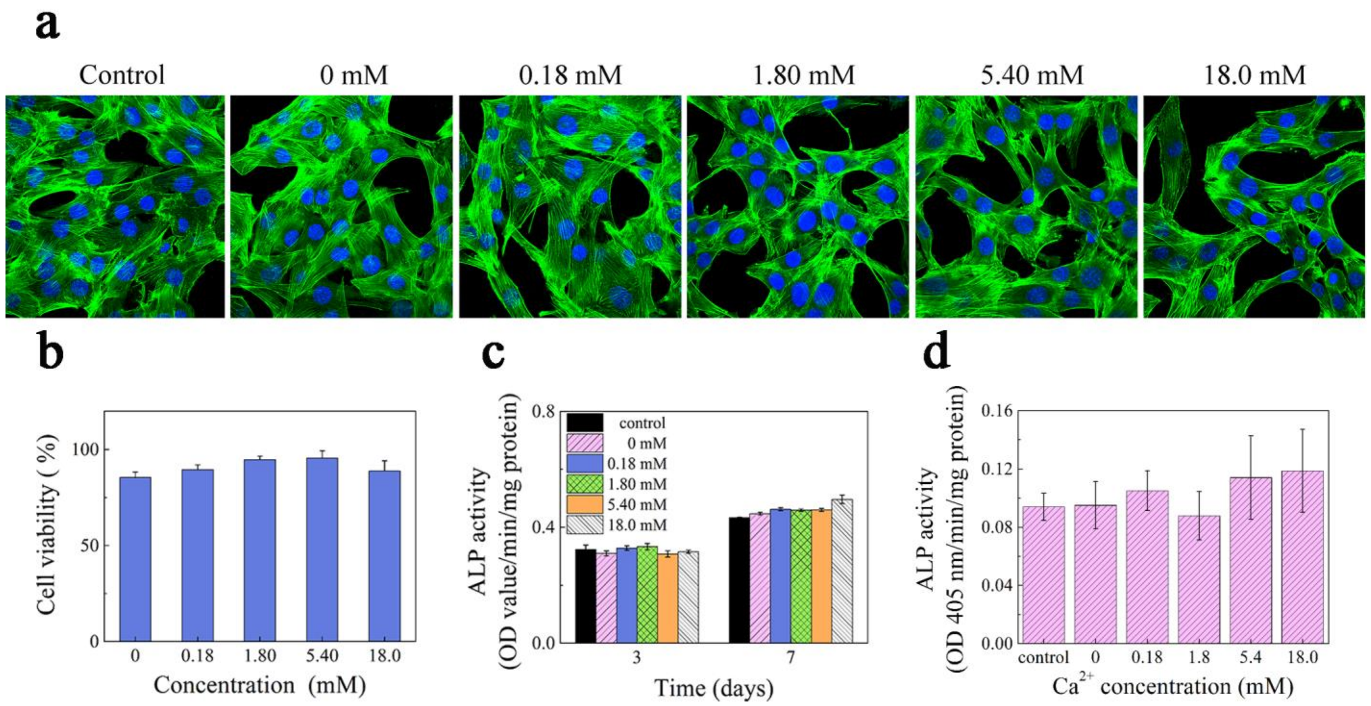

Fig S3 C2C12 response to Ca^{2+} alone at various concentrations. (a) Cell morphology after exposure to elevated concentration of Ca^{2+}. Here, C2C12 cells were first exposed to rhBMP-2-containing specific DMEM medium for 6h and then transferred into regular DMEM for another 66 h. Cell nucleus was stained with DAPI (blue) and actin skeleton was stained with phaloidin (green). (b) The effect of Ca^{2+} on the cell proliferation. Cell culture was the same as the above cell morphology. MTT assay was applied for investigating the cell proliferation by calcium. $p > 0.05$. (c) ALP activity measurement in the non-osteogenic medium. Cells were treated by elevated concentration of calcium ions (0, 0.18, 1.80, 5.40 and 18.0 mM) as the above-mentioned two-stage culture process. (d) ALP activity measurement in the osteogenic media. Cells were treated by elevated concentration of calcium ions in specific DMEM medium containing ascorbic acid, beta-glycerophosphate, and dexamethasone for 6h and then cultivated with normal DMEM for another 66h. No significant difference was observed. ($n=5$, $p>0.05$).

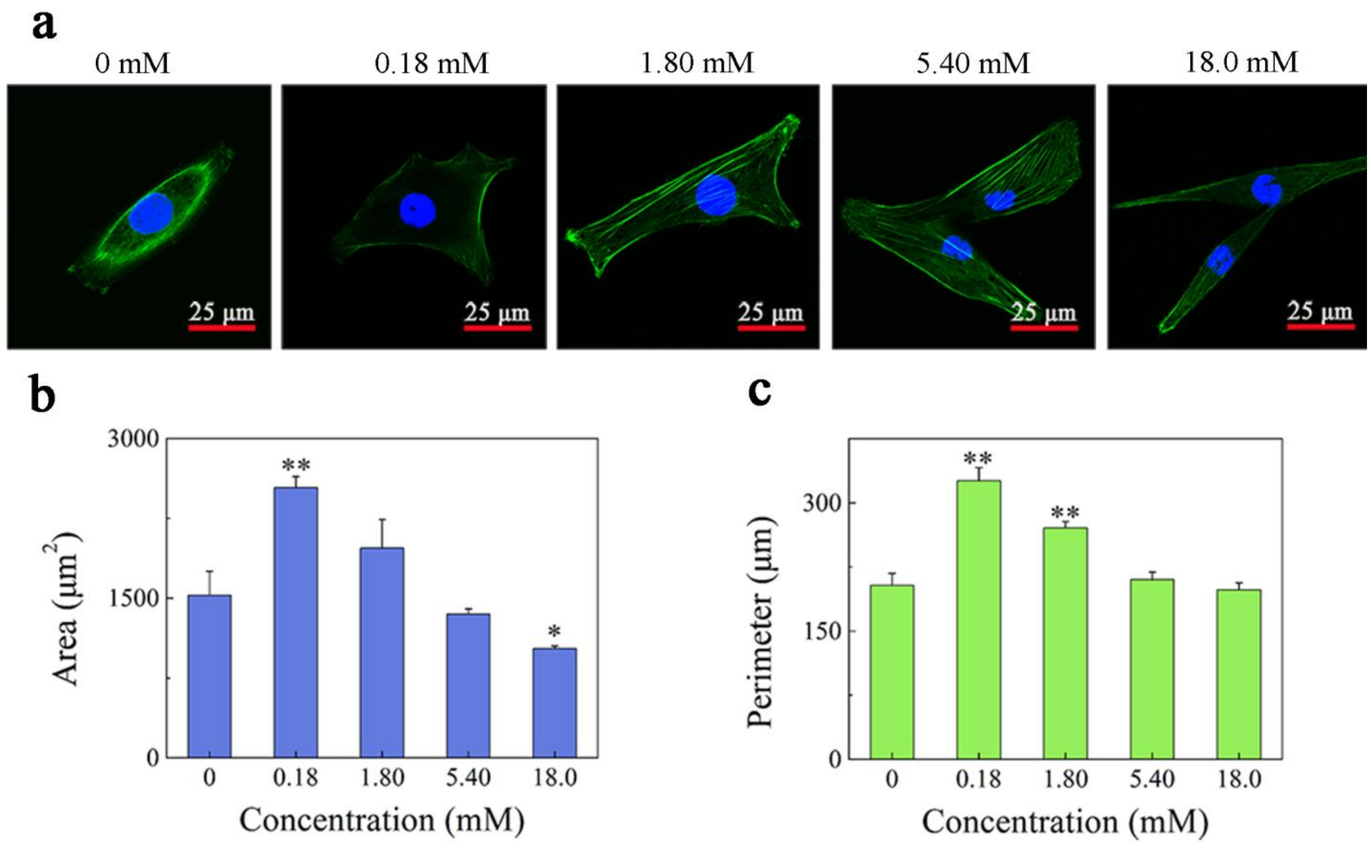

Fig S4 Cell morphologies after cultured with rhBMP-2 with different concentrations of calcium ions. (a) C2C12 cells treated with elevated [Ca^{2+}] and rhBMP-2 in specific DMEM for 6 h and stained with DAPI (blue) for cell nucleus and phaloidin (green) for actin skeleton. Scale bar is 25 μm. Summarized cell area and shape parameters corresponding to C2C12 cultured in different media, including Area (b) and perimeter (c) (*$p < 0.05$, **$p < 0.01$, compared with free rhBMP-2).

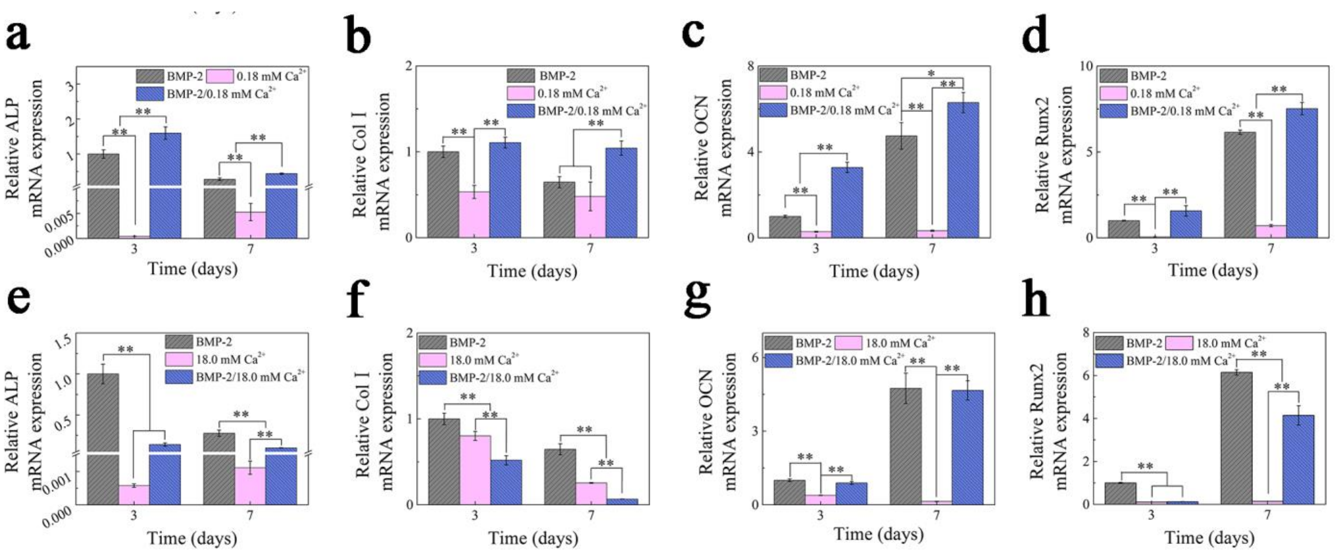

Fig S5 Gene expression. (a-d) Expression levels for genes ALP (a), Col (b), OCN (c) and Runx2 (d) from cells incubated with 3 different media (free rhBMP-2, 0.18 mM calcium ions alone or in combination) for 3 and 7 days. (e-h) Expression levels for genes ALP (e), Col (h), OCN (g) and Runx2 (h) from cells incubated with 3 different media (free rhBMP-2, 18.0 mM calcium ions alone and 18.0 mM calcium ions/rhBMP-2) for 3 and 7 days. (n = 3, $*p < 0.05$, $**p < 0.01$, compared with free rhBMP-2). Cells were cultured in rhBMP-2 (2.0 μg/mL) media with various calcium in specific DMEM for 6 h, and then exposed to normal DMEM without rhBMP-2 for another 66h and 162h, respectively. Compared with rhBMP-2-contained groups, the osteo-related genes were barely expressed in only calcium-added media.

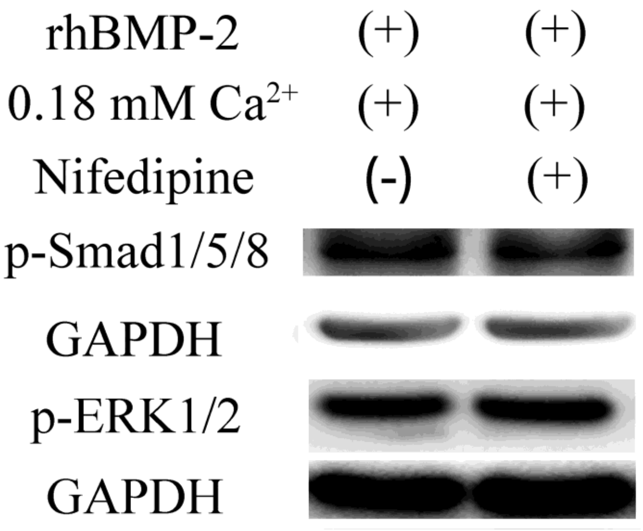

Fig S6 Western blot analysis for the effect of Ca^{2+} channel on the Smad and ERK1/2 pathway. For the blocking calcium channel, C2C12 cells were pretreated with nifedipine (20 μM) for 30 min followed by stimulation with 0.18 mM Ca^{2+}/rhBMP-2 in 2% FBS-containing specific DMEM for 3h. Otherwise, the cells were directly exposed to 0.18 mM Ca^{2+}/rhBMP-2 in 2% FBS-containing specific DMEM for 3h. Cell lysates were analyzed by Western blotting in the same way as mentioned before cells exposure to 2.0 μg/mL rhBMP-2

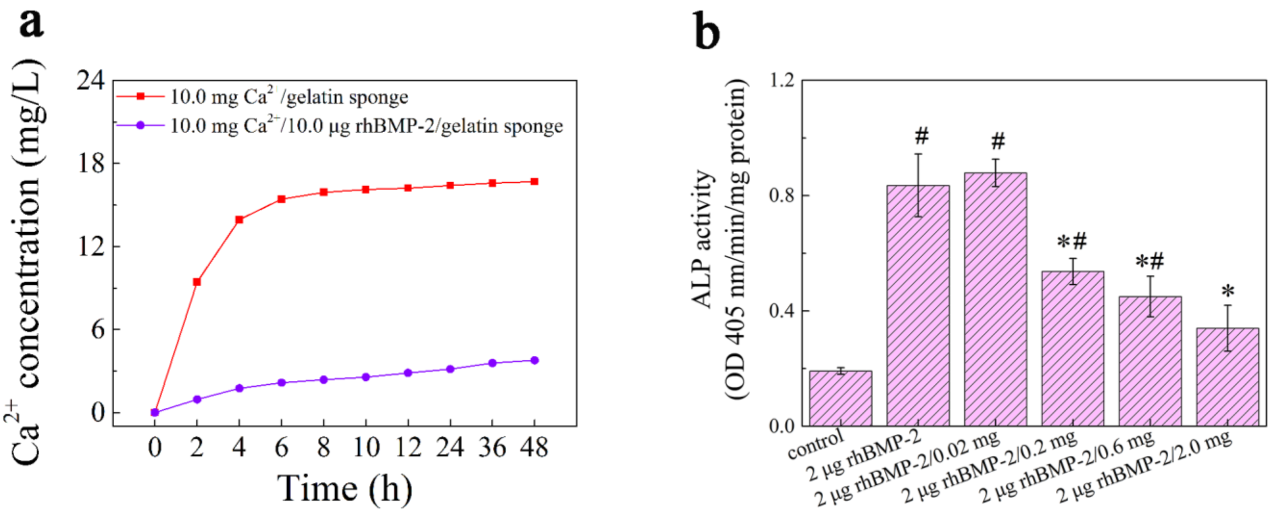

Fig S7 (a) Release kinetic of calcium from the gelatin sponge used for the *in vivo* studies. Gelatin sponge contained 10.0 mg $CaCl_2$ or 10.0 mg $CaCl_2$/10.0 μg rhBMP-2 were prepared as mentioned in "Materials and methods" and then immersed in 200 mL specific media (without calcium). After indicated time, the sponge was removed and the culture medium was collected. The concentration of Ca^{2+} in medium was analyzed with an inductively coupled plasma atomic emission spectrometer (ICP-AES). (b) ALP activity in 3D by injecting calcium and rhBMP-2 in gelatin sponge to mimic the in vivo procedure. The C2C12 cells were seeded in 24-well plates in 1 mL DMEM containing 10% FBS for 24 h incubation. The gelatin sponge was prepared as mentioned before. The concentration of rhBMP-2 in vivo was much higher than that in vitro. Therefore, the sponge contained 2 μg rhBMP-2 (comparable with 2.0 μg/mL in previous experiment) for 3D experiment in vitro. The mass of calcium in the 3D scaffold were 0.02, 0.2, 0.6 and 2.0 mg, respectively.

Table S1 The changes of frequency for BMPIB and BMPRII adsorption after preadsorption of rhBMP-2, $[Ca^{2+}]_L$/rhBMP-2 and $[Ca^{2+}]_H$/rhBMP-2

	Δf (Hz)		
	**BMPRIA**	**BMPRIB**	**BMPRII**
**free rhBMP-2**	14.44	5.86	2.99
**$[Ca^{2+}]_L$/rhBMP-2**	18.92	5.93	3.12
**$[Ca^{2+}]_H$/rhBMP-2**	9.12	4.98	2.71

Table S2 Parameters of primers utilized for detecting osteogenetic gene expression

Gene	Direction	Sequence(5’-3’)
**ALP**	Forward	CCA ACT CTT TTG TGC CAG AGA
	Reverse	GGC TAC ATT GGT GTT GAG CTT TT
**Collagen I**	Forward	GGT ATG CTT GAT CTG TAT CTG C
	Reverse	AGT CCA GTT CTT CAT TGC ATT
**Osteocalcin**	Forward	CTG ACA AAG CCT TCA TGT CCA A
	Reverse	GCG GGC GAG TCT GTT CAC TA
**Runx2**	Forward	CGG CCC TCC CTG AAC TCT
	Reverse	TGC CTG CCT GGG ATC TGT
**GAPDH**	Forward	GTC GTG GAG TCT ACT GGT GTC
	Reverse	GAG CCC TTC CAC AAT GCC AAA

References

1 Rusmini, F., Zhong, Z. & Feijen, J. Protein immobilization strategies for protein biochips. *Biomacromolecules* **8**, 1775-1789 (2007).

2 Frostell-Karlsson, Å. *et al.* Biosensor analysis of the interaction between immobilized human serum albumin and drug compounds for prediction of human serum albumin binding levels. *J Med Chem* **43**, 1986-1992 (2000).
